# Artificial Intelligence-Based Mitosis Detection in Breast Cancer Histopathology Images Using Faster R-CNN and Deep CNNs

**DOI:** 10.3390/jcm9030749

**Published:** 2020-03-10

**Authors:** Tahir Mahmood, Muhammad Arsalan, Muhammad Owais, Min Beom Lee, Kang Ryoung Park

**Affiliations:** Division of Electronics and Electrical Engineering, Dongguk University, 30 Pildong-ro 1-gil, Jung-gu, Seoul 04620, Korea; tahirmahmood.cs@gmail.com (T.M.); arsal@dongguk.edu (M.A.); malikowais266@gmail.com (M.O.); mblee@dongguk.edu (M.B.L.)

**Keywords:** breast cancer, mitotic cell count, artificial intelligence, Faster R-CNN, deep CNNs

## Abstract

Breast cancer is the leading cause of mortality in women. Early diagnosis of breast cancer can reduce the mortality rate. In the diagnosis, the mitotic cell count is an important biomarker for predicting the aggressiveness, prognosis, and grade of breast cancer. In general, pathologists manually examine histopathology images under high-resolution microscopes for the detection of mitotic cells. However, because of the minute differences between the mitotic and normal cells, this process is tiresome, time-consuming, and subjective. To overcome these challenges, artificial-intelligence-based (AI-based) techniques have been developed which automatically detect mitotic cells in the histopathology images. Such AI techniques accelerate the diagnosis and can be used as a second-opinion system for a medical doctor. Previously, conventional image-processing techniques were used for the detection of mitotic cells, which have low accuracy and high computational cost. Therefore, a number of deep-learning techniques that demonstrate outstanding performance and low computational cost were recently developed; however, they still require improvement in terms of accuracy and reliability. Therefore, we present a multistage mitotic-cell-detection method based on Faster region convolutional neural network (Faster R-CNN) and deep CNNs. Two open datasets (international conference on pattern recognition (ICPR) 2012 and ICPR 2014 (MITOS-ATYPIA-14)) of breast cancer histopathology were used in our experiments. The experimental results showed that our method achieves the state-of-the-art results of 0.876 precision, 0.841 recall, and 0.858 F1-measure for the ICPR 2012 dataset, and 0.848 precision, 0.583 recall, and 0.691 F1-measure for the ICPR 2014 dataset, which were higher than those obtained using previous methods. Moreover, we tested the generalization capability of our technique by testing on the tumor proliferation assessment challenge 2016 (TUPAC16) dataset and found that our technique also performs well in a cross-dataset experiment which proved the generalization capability of our proposed technique.

## 1. Introduction

**Figure 1:**
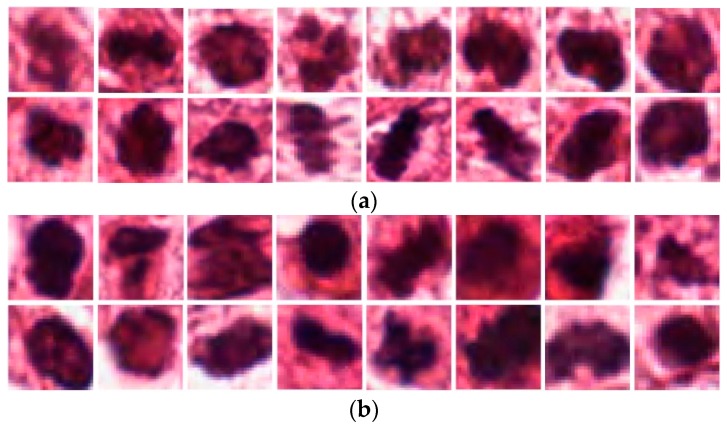
Examples of (**a**) mitotic and (**b**) non-mitotic cells.

Breast cancer is the most common and leading cause of death among women. According to the global cancer project (GLOBOCAN 2012), breast cancer accounts for 25.1% of all cancers in women [1]. Early diagnosis of breast cancer is an important factor for the reduction of the mortality rate because its treatment plan is advised on the basis of the grade and prognosis of the cancer. To determine the grade of breast cancer, the Nottingham grading system has been widely used. According to this system, there are three biomarkers for the grading of breast cancer in histopathology images. These biomarkers are nuclear atypia, tubule formation, and the mitotic cell count. Among these biomarkers, the mitotic cell count is the most important biomarker as the mitosis cell division process is directly related to the prognosis of tumors [2]. In practice, mitotic cells are generally detected via the visual inspection of the histopathology slide images of the breast under high-resolution microscopes. However, this procedure is tedious, time consuming, and subjective. A low-skilled pathologist could thus arrive at inaccurate detections, which could have serious consequences. Recently, artificial-intelligence (AI) techniques had a great impact on every field of life and even in the medical field. The majority of the processes are now automated and can even be used as a second-opinion system in medical diagnosis. AI techniques [3,4,5,6,7,8,9,10] have been developed previously for solving problems in the medical field. Mitotic-cell detection can also be automated using AI techniques; however, it comprises several challenges. For example, it is difficult to differentiate between mitotic and normal cells without pathological knowledge and the use of high-resolution microscopes because mitotic cells have a texture and morphological features that are similar to normal cells, as shown in Figure 1. Moreover, some of the other organelles of the cell, such as apoptotic cells, have a similar appearance to that of mitotic cells. The mitosis process comprises four stages where each has its own unique characteristics, and thus, a robust technique is required to be developed for detecting diverse mitotic cells. Another major challenge is the maintenance of the standard data-preparation environment. Biopsy, slide preparation, and scanning procedures are required to be performed carefully because a low performance is obtained in the case of issues in data collection, slide preparations, and scanning [11].

Mitotic-cell detection techniques can be categorized into two divisions based on the features extracted from regions of interest (ROIs): handcrafted features and deep features. Handcrafted features are extracted from ROIs by using conventional image-processing techniques. Features such as color, morphology, and texture are extracted, which is followed by classification using machine-learning classification algorithms such as an artificial neural network and a support vector machine (SVM). Previous research [12,13,14,15,16] that has been conducted on this approach has demonstrated good performance and can be used in small-scale applications. In the second approach, deep features are extracted from ROIs by using deep-learning techniques [17,18,19,20]. In the mitotic-cell detection task, deep features-based techniques are further divided into three main categories based on the problem formulation. Some researchers consider mitotic-cell detection as a classification task, while others consider it as a semantic segmentation task because of the pixels-based annotations. Few others also consider it to be an object-detection task because the objective was not to determine the shape of the mitotic cells but to count them. In our proposed work, we considered mitotic-cell detection as an object-detection task and proposed a technique that provides the state-of-the-art results.

The rest of this paper is organized as follows. Section 2 and Section 3 present the related research works and our contributions, respectively. Section 4 and Section 5 respectively present the proposed method and explanations of the experimental setup and performance analysis. Section 6 presents a discussion of the obtained results, while Section 7 presents the conclusion of our research.

## 2. Related Works

Mitotic-cell detection in hematoxylin-and-eosin-stained (H&E-stained) biopsy images have been researched since the invention of whole-slide imaging scanners. In addition, owing to recent developments in AI, a number of techniques have been developed which demonstrate outstanding performance and can be used in real-time applications. Previous research can be divided into two categories: handcrafted-features-based and deep-features-based research. Details of each of the aforementioned categories are provided in the following section.

### 2.1. Mitosis Detection Using Handcrafted Features

Conventional image-processing techniques have been used for the extraction of handcrafted features such as shape, texture, and color, which are followed by the use of machine-learning algorithms for mitotic cell detection. In the extant literature, several techniques comprising the use of handcrafted features have been presented. Irshad presented a technique [12] in which all the expected objects were first segmented, and statistical and morphological features were extracted and classified using a decision-tree classifier [21]. This technique ranked second in the mitosis detection challenge of the international conference on pattern recognition (ICPR) 2012. Tashk et al. presented a technique [13] based on local binary pattern (LBP) and SVM [21] as a classification algorithm. LBP features have high discriminative power and are also invariant to grayscale changes. This technique ranked third in the aforementioned ICPR 2012 challenge. Sommer et al. used shape and intensity features along with texture features in their proposed technique [14] to distinguish between mitotic and non-mitotic cells while using an SVM classifier. They used two open-source biomedical image analysis software: “ilastik” [22] for the segmentation of objects and “CellCognition” [23] for classification into mitotic and non-mitotic cells. This technique comprises a small amount of parameter tuning and no user effort because open source software are used. However, this technique demonstrates a relatively low detection performance as compared to other handcrafted feature-based techniques. Paul et al. focused on the nucleus of the cell in their proposed technique [15]. They used a regenerative random forest tree classifier that demonstrated an excellent performance. However, this technique requires significant computational resources, and thus, it cannot be used in practical clinical application. The majority of handcrafted-features-based methods as presented in Table 1 provide a low detection performance as compared to the recently developed deep-features-based techniques, and they are also computationally expensive owing to the conventional image-processing operations required for the segmentation of objects.

### 2.2. Mitosis Detection Using Deep Features

A deep-features-based method is more powerful than a handcrafted-features-based method because it takes into consideration thousands of meaningful features during the training. Ciresan et al. presented a technique [17] based on the sliding window approach for the extraction of deep features from images. This technique ranked first in the ICPR 2012 mitosis-detection contest. The sliding window approach is computationally expensive, and thus, this technique is not suitable for clinical application. Malon et al. combined handcrafted nuclear features and deep features from a convolutional neural network (CNN) [18]. This technique also comprised the use of an additional CNN that reduces the sensitivity of mitotic cells during feature extraction; however, it demonstrates a low performance and high computational complexity. Wang et al. presented a cascaded technique [19] in which two classifiers were used independently. One classifier is trained with handcrafted features and the other is trained with CNN features. In the testing stage, a third classifier is used if the outputs of the two classifiers are different. This technique is fast and computationally inexpensive; however, the ROI-selection performance with conventional image processing is lower than that obtained with the deep-learning technique. Chen et al. presented a two-stage technique [20] in which mitotic cells were segmented by a fully convolutional network (FCN) in stage 1, while in stage 2, all the detected objects were further refined by an additional CNN. Recently, region-based CNNs performed well in a number of computer vision applications. For mitotic-cell detection, Li et al. presented a technique [2] based on Faster region convolutional neural network (Faster R-CNN) [28] and residual network (Resnet)-50 [29]. Faster R-CNN initially detects mitotic cells, which are further refined by Resnet-50. The Faster R-CNN used in this technique comprises visual geometry group (VGG)-16 [30] as a feature-extraction network. This technique provides good results and requires less inference time; however, Faster R-CNN results can be improved by using other feature-extraction networks because VGG-16 has the vanishing gradient issue. Li et al. also presented another technique [24] based on concentric circles for a weakly annotated dataset of ICPR 2014. This approach is good for weakly annotated datasets. Cai et al. used a modified Faster R-CNN in their proposed technique [25]. Resnet-101 was used for the feature extraction of the Faster R-CNN. Although this technique is good, Resnet-101 can be replaced by a shallow network. Li et al. presented a lightweight region-based CNN technique [26] that was developed using standard desktop computers without graphics processing units (GPUs). Based on the mask R-CNN [31], Dodballapur et al. presented a technique [27] and that comprised Resnet-50 as a feature-extraction network. Xception network [32] was used for the reduction of false positives. This technique provides high accuracy; however, owing to its use of expensive GPUs and intensive training, it is not suitable for use in practical clinical applications. Table 1 presents a comparison between existing methods and proposed method for mitotic cell detection.

## 3. Contribution

The major contributions of this work are summarized as follows:

- This proposed technique provides state-of-the-art results in mitosis-detection tasks as per the ICPR 2012 and ICPR 2014 contest datasets.
- Faster R-CNN is used in the first stage in which the primary detection of mitotic cells was performed. We adopt Resnet-50 as a features-extraction network for the first time, thus obtaining better results as compared to the other techniques.
- In the proposed technique, a large number of false positives are produced because of the minute differences between mitotic and non-mitotic objects. To reduce the number of false positives, we perform post-processing on the basis of statistical, texture, shape, and color features.
- To further reduce the number of false positives, we perform a score-level fusion of Resnet-50 and a dense convolutional network (Densenet)-201. This is used for the first time in the mitotic-cells-detection task and it significantly reduces the number of false positives.
- - To allow other researchers to perform fair comparisons, our trained models are publicly available in [33].

## 4. Proposed Method

### 4.1. Overview of Proposed Approach 

**Figure 2:**
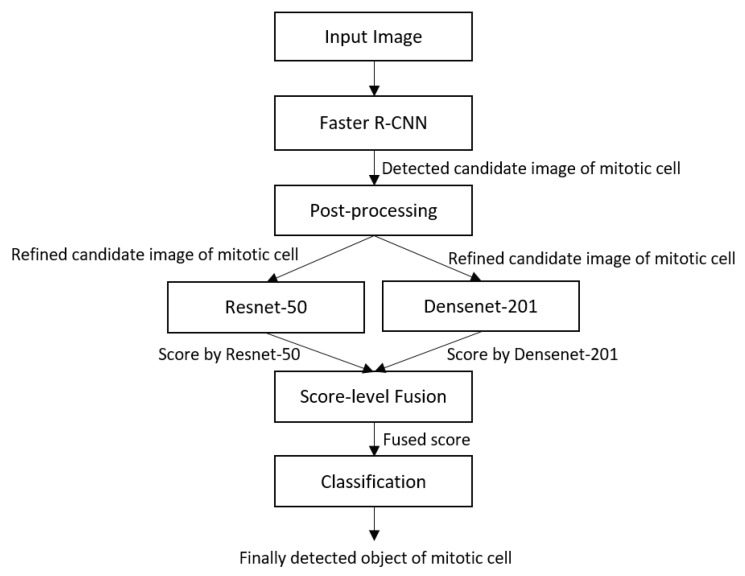
Flow diagram of the proposed technique. Faster R-CNN, Faster region convolutional neural network.

Our proposed technique comprises a multistage mitotic-cell detection framework. There are four main stages in the proposed technique. In stage 1, an image is input into the trained Faster R-CNN detector, which is trained on the training datasets of the ICPR 2012 and ICPR 2014 mitosis-detection data. The detection results obtained using Faster R-CNN are adversely affected by a large number of false positives. Therefore, further post-processing is performed to reduce the number of false positives in stage 2 on the basis of statistical, texture, shape and color features. In stage 3, independently trained Resnet-50 and Densenet-201 scores are fused, and the final classification of the mitotic and non-mitotic cells is performed in stage 4. Figure 2 presents the flow diagram of the proposed technique.

### 4.2. Mitotic-Cell Detection Using Faster R-CNN

**Figure A1:**
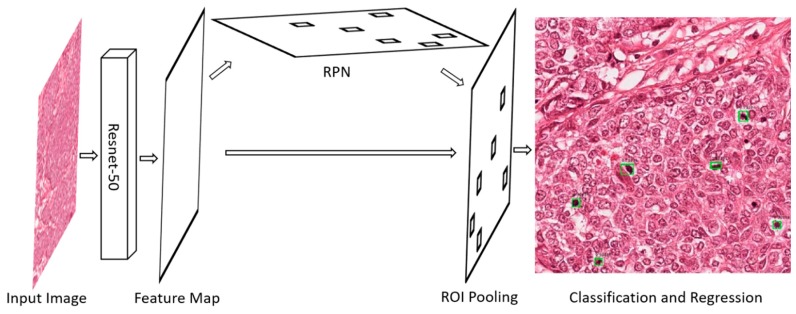
Faster R-CNN architecture; the feature map is extracted from the input image using the Resnet-50 feature-extraction network followed by the generation of region proposals in the RPN and the final mitotic-cells detection in the classification network [28].

The image is input into the Faster R-CNN trained network for the initial mitotic-cell detection. The Faster R-CNN is a region-based CNN [28] that was presented in the object-detection competition of the ImageNet Large-Scale Visual Recognition Challenge 2015 [34]. The Faster R-CNN is the combination of three sub-networks, the feature-extraction network, region proposal network (RPN) [35], and classification network, as shown in Figure A1 in the appendix. In the feature-extraction network, a feature map known as an activation map is generated, and appropriate deep features are extracted. Different types of CNNs can be used as a feature extractor depending on the application requirements, and we use a Resnet-50 pre-trained on ImageNet database as a feature extraction network. Table A1 in the appendix presents the detailed architecture of the Resnet-50. The Resnet-50 includes 50 weighted layers that are based on the idea of skipping the blocks of convolution layers by using shortcut connections. In general, the basic blocks known as “bottleneck” blocks follow two design rules: use the same number of filters for the same output feature size and double the filters if the feature size is halved. Moreover, the down-sampling is performed by the convolution layers with a stride of 2, and batch normalization is performed after each convolution and before the rectified-linear-unit (ReLU) activation. An identity short-cut is used if the input and output have the same dimensions, and the projection shortcut is used to match the dimensions through 1 × 1 convolutions if the dimensions increase [29]. The feature map extracted from the last convolutional layer is forwarded to the RPN and classification networks (ROI pooling network). As only the feature map from the feature-extraction network is necessary for the RPN and classification network instead of the final classification, therefore, only the 49 convolutional layers without average pooling and fully connected layers in Resnet-50 are used in the feature-extraction network, as shown in Table A1 in the appendix.

The RPN is the second part of the Faster R-CNN. It is responsible for the generation of region proposals of various sizes and ratios, which are used in the final classification network. The detailed network architecture is presented in Table A2 in the appendix. In the RPN, anchor boxes of different scales and aspect ratios are initially generated over each pixel of the feature map. In general, nine anchor boxes with scales of 128, 256, and 512 and aspect ratios of 1:1, 1:2, and 2:1 are used. RPN predicts the probability that an anchor box is an object or background. The final list of the proposals is filtered according to the intersection over union (IOU) threshold of 0.8 and non-maximum suppression (NMS) for the target objects. The list of filtered anchor boxes at this stage is the required object proposals, which is forwarded to the next stage. The transformation of the anchor boxes to the final predicted region proposals requires the use of the following Equations (1) and (2). Equation (1) presents the scale invariant translation between the center coordinates, while Equation (2) presents the log-space translation between the width and height.

(1) $$v_{x}=\frac{x_{p}-x_{a}}{w_{a}},\;v_{y}=\frac{y_{p}-y_{a}}{h_{a}}$$

(2) $$v_{w}=\log\left(\frac{w_{p}}{w_{a}}\right),\;v_{h}=\log\left(\frac{h_{p}}{h_{a}}\right)$$

where $v_{x},\;v_{y},v_{w},\;\mathrm{and}\;v_{h}$ are the bounding box regression vectors, and x,y,w,and h are the x and y coordinates of the center, width, and height of each box, respectively. Moreover, $x_{p}\;\mathrm{and}\;\;x_{a}$ are the center coordinate x of each proposal box and anchor box, respectively.

The feature map extracted in step 1 and the region proposals generated in step 2 are input into the classification part of the Faster R-CNN. Table A3 in the appendix presents the details of the classification network used in the Faster R-CNN. In the classification network, the feature map is cropped at a specific point by using region proposals. Each of the cropped feature maps has a different size, and therefore, ROI pooling is applied to obtain a uniform size. The bounding box regression vectors and mitotic cell probabilities are obtained after passing through fully connected layers. The bounding box regression vectors are used for the refinement of proposal boxes into prediction boxes followed by the removal of overlapping boxes via NMS, which results in the final detection results. We use the Faster R-CNN because it provides the highest accuracy and lowest computational complexity as compared to other region-based CNNs such as R-CNN [36] and Fast R-CNN [37]. In R-CNN and Fast R-CNN, region proposals are generated by a selective search algorithm [38] followed by the use of a detection network for classification and bounding boxes regression. The selective search algorithm and detection network are decoupled because false negatives have a direct effect on the detection network. Another disadvantage is the high computational cost and required time of the selective search algorithm. To overcome these shortcomings, the Faster R-CNN is used to replace the selective search by the RPN and shares convolutions across region proposals. Therefore, the Faster R-CNN is computationally inexpensive and has a high accuracy.

In the proposed technique, the Faster R-CNN detects the mitotic cells. During training, the weights are trained to get a minimal loss for each anchor box or proposal in the mini-batch. The loss function of the Faster R-CNN is as follows:

(3) $$L\left(p,p^{*},v,\;v^{*}\right)=L_{cls}\left(p,p^{*}\right)+\sigma p^{*}L_{reg}\left(v,v^{*}\right)$$

In Equation (3), p is the probability that an anchor box is the object. $p^{*}$ is the ground truth label (mitotic cell = 1, and background = 0), while v and $v^{*}$ are the bounding-box regression vector of the anchor box and its corresponding ground truth, respectively. Similarly, $L_{cls}$ indicates the classification loss function, and $L_{reg}$ represents the regression loss function. σ is the weight-balancing parameter used for the weighting of cls and reg. During the training, the weights are trained to minimize the loss value, and the Faster R-CNN can accurately predict the position of the mitotic cells.

### 4.3. False-Positive Mitotic-Cells Removal via Post-Processing

Post-processing comprises the second stage of our proposed technique. In this stage, the mitotic cells detected using the Faster R-CNN are refined on the basis of handcrafted features. After the analysis of the mitotic cells, we observe that there is no appropriate distribution of gray levels. Instead, the mitotic cells usually have a dark blue color with an irregular texture. Therefore, we focus on color and texture in the post-processing. We use statistical LBP [39] histograms of oriented gradients (HOG) [40], and color features for the post-processing. Statistical features have two main sub-divisions: first order and second order. The first order statistical features are related to the gray-level distribution in the image. Single pixel-based estimation is performed to extract in the first order statistical features, while the spatial relationship between the pixels is ignored. Standard deviation, skewness, and kurtosis are examples of first-order statistical features. Histogram-based features are also included in this category. In second-order statistical features, the spatial relationship between the pixels is considered. Co-occurrence or run-length matrices are used for the extraction of the features. Angular second moment, contrast, correlation, homogeneity, and entropy are examples of second-order statistical features.

In our method, we use the first-order statistical features of mean, standard deviation, skewness, and kurtosis. The mean of an image describes the average color of an image, while the standard deviation is the estimation of the underlying brightness probability distribution. Skewness is the description of the darker and lighter colors with respect to the mean, and kurtosis provides information of the uniformity distribution of the intensity distribution. In our method, there is a minute difference between the statistical features of the cells. The optimal threshold is obtained with the training data for each of the features, and each of the detected objects is compared with the threshold for the acceptance or rejection as a mitotic cell. The same procedure is iterated with the remaining post-processing based on the second, third, and fourth features.

The second feature used for the post-processing is the LBP. It is the measure of the local image contrast [41], and it has been used in a number of computer vision applications [42,43,44]. For the extraction of the LBP feature, each center-pixel value is subtracted from each of the eight adjacent pixels. If the result of the subtraction yields a negative number, the eight adjacent pixels are represented by 0, else it is represented by 1. For labeling this specific pixel, a decimal value is obtained from the conversion of binary code obtained from the concatenation of all the binary codes in a clockwise direction starting from the top left adjacent pixel. Equations (4) and (5) represents the extraction process of the LBP features.

(4) $$LBP_{R,P}=\sum_{i=0}^{P-1}s\left(g_{i}-g_{c}\right)2^{i},$$

(5) $$where\;s\left(x\right)=\{\begin{array}{c} 1,\;if\;x\geq 0\\ 0,\;if\;x<0 \end{array}$$

In the above equation, P represents the number of neighboring pixels, R is the radius of the LBP circle, and $g_{i}$ and $g_{c}$ are the neighboring pixels and center-pixel gray levels, respectively. The binary value is represented by s(x). In our method, the LBP features play an important role because the mitotic cells have an irregular texture and can be differentiated from the normal cells based on the contrast of the ROIs. Therefore, the LBP features are used in the post-processing.

**Figure A2:**
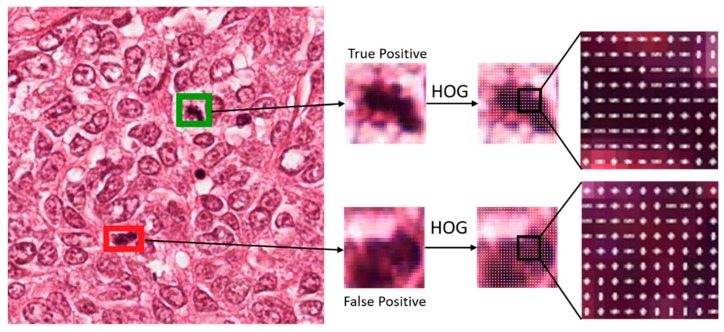
HOG features from true-positive (green box) and false-positive (red box) images.

The third post-processing feature is HOG. It is a shape-based feature [45] and has been used in various computer vision applications [46,47]. The target image is pre-processed and resized to a ratio of 1:2 because the image is required to be divided into 8 × 8 or 16 × 16 patches. The gradient vector of each pixel is calculated along with its magnitude and direction. If we divide the image into cells of 8 × 8 pixels, the magnitude of 64 pixels in each cell is binned and added into nine buckets of unsigned direction. A 2 × 2-cells block then slides over the image. For each block, four histograms of four cells are concatenated into one dimensional vector of 36 values followed by normalization to obtain a unit weight. The concatenation of all the block vectors is the final HOG feature. Figure A2 in the appendix presents the HOG feature for the mitotic cells. The last set of features includes the color features. A color histogram is used for the extraction of the RGB colors because there exists a diversity in the colors in mitotic cells. In the color histogram, the frequencies of different colors in the image are represented by the discretization of the color values into a number of bins. The frequency of each color in a bin is then represented by the histogram.

Feature extraction is followed by the acceptance or rejection of objects as a mitotic cell based on an optimal threshold of each of the feature. These optimal thresholds are experimentally obtained from the training data. Objects are accepted or rejected as a mitotic cell by comparing with thresholds set for each of the feature. In detail, any candidate is determined as a mitotic cell in the case that its feature value (for example, HOG, LBP, statistical, and color features) is higher than the optimal threshold whereas it is determined as non-mitotic cell if its value is lower than the threshold. Different set of features and thresholds are tried to get the best discriminative rule for the acceptance of a large number of mitotic cells.

### 4.4. Final Classification of Mitotic Cells via Score-Level Fusion of Two CNNs

In the next stage, the final classification of the mitotic cells is performed via the score-level fusion of Resnet-50 and Densenet-201. The detailed structure of Resnet-50 is presented in Table A1 in the appendix, while the explanations of Resnet-50 are presented in Section 4.2. As the final score should be obtained in this case, average pooling, Softmax, fully connected, and classification layers are included in Resnet-50 in Table A1. Densenet adopts a dense connectivity, which improves the skip connection structure of Resnet. This is the method of concatenating the feature maps of the *l*th layer and previous layers based on dense block. Therefore, the input of the *l*th layer comprises the concatenated feature map of the previous layers ($x_{0},x_{1},\ldots ,x_{l-1})$, as presented in Equation (6) [48]. In Equation (6), $H_{l}\left(.\right)$ is a function that includes the operations of convolution, pooling, batch normalization (BN), or ReLU. The detailed structure and explanations of Densenet-201 can be referred to in [48].

(6) $$x_{l}=H_{l}\left(\left[x_{0},x_{1},\ldots ,x_{l-1}\right]\right)$$

Score-level fusion is an integration technique in which the scores from multiple modalities are fused to make a decision. In general, the data of a single modality lack uniqueness and non-universality and also comprise noise [49]. Therefore, multi-modal data obtained by the fusion of single modalities have better discrimination abilities and are used in various applications [50,51]. The fusion of the information can usually be performed at three levels: (a) feature-extraction level, (b) matching-score level, and (c) decision level. In the feature-extraction level, a higher-dimension feature vector is obtained by concatenating the features that are obtained from the individual classifier. Feature-reduction techniques are employed for the selection of useful features. The score-level fusion integrates the classifier’s scores based on the proximity of the scores, and the decision-level fusion is performed based on the final decision of “Yes” or “No” [52].

**Figure A3:**
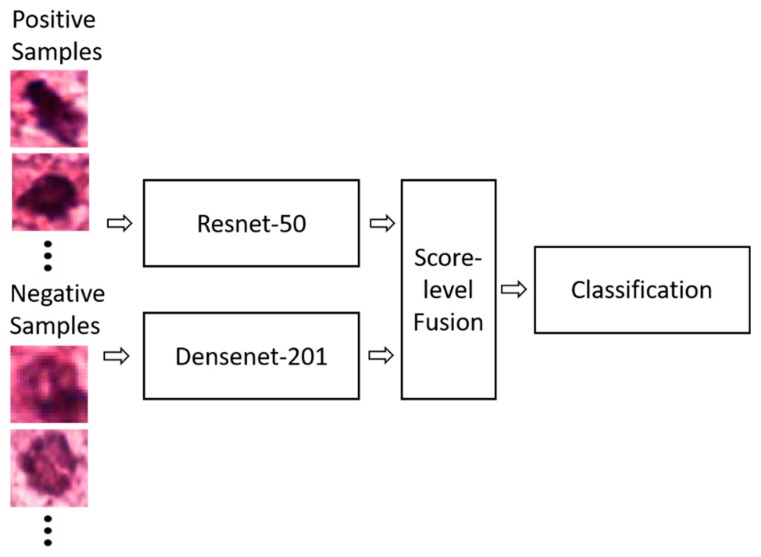
Score-level fusion of Resnet-50 and Densenet-201 and classification of mitotic cells.

In our work, we have used the score-level fusion technique because of its superior performance as compared to other fusion techniques [52]. The pretrained Resnet-50 and Densenet-201 are each trained over the patches of the detected mitotic cells. In the testing phase, an image is passed through trained Faster R-CNN and each of the detected objects are then passed through trained Resnet-50 and Densenet-201 networks. We know that probabilistic scores are produced from the output layer, so we obtained that score for each of the classifiers and fused it together for final classification. In score-level fusion the match scores are fused together to render a decision about the identity of object. Moreover, as mentioned above for different levels of fusion, we adopted hierarchical score-level fusion. In detail, the score of input candidate is compared with a predetermined threshold in the first hierarchical stage, and that whose score is larger than the threshold is determined as mitotic cell. Then, it is compared with the threshold in the second stage and proceeds until the last stage. After all the stages, the accepted one is determined as a mitotic cell. These thresholds were experimentally determined with training data. It can be seen in Figure A3 of the appendix how positive and negative samples are passed through Resnet-50 and Densenet-201, followed by the score-level fusion and classification.

## 5. Experiments and Performance Analysis

In this section, we present the experimental datasets and environment settings with hardware and software specifications used for performing the experiments. Moreover, the evaluation criteria and performance analysis are also described in detail.

### 5.1. Datasets

In the proposed technique, two publicly available datasets of mitotic-cell detection in histopathology images are used. The details of each dataset are as follows.

#### 5.1.1. ICPR 2012 MITOSIS Dataset

**Figure 3:**
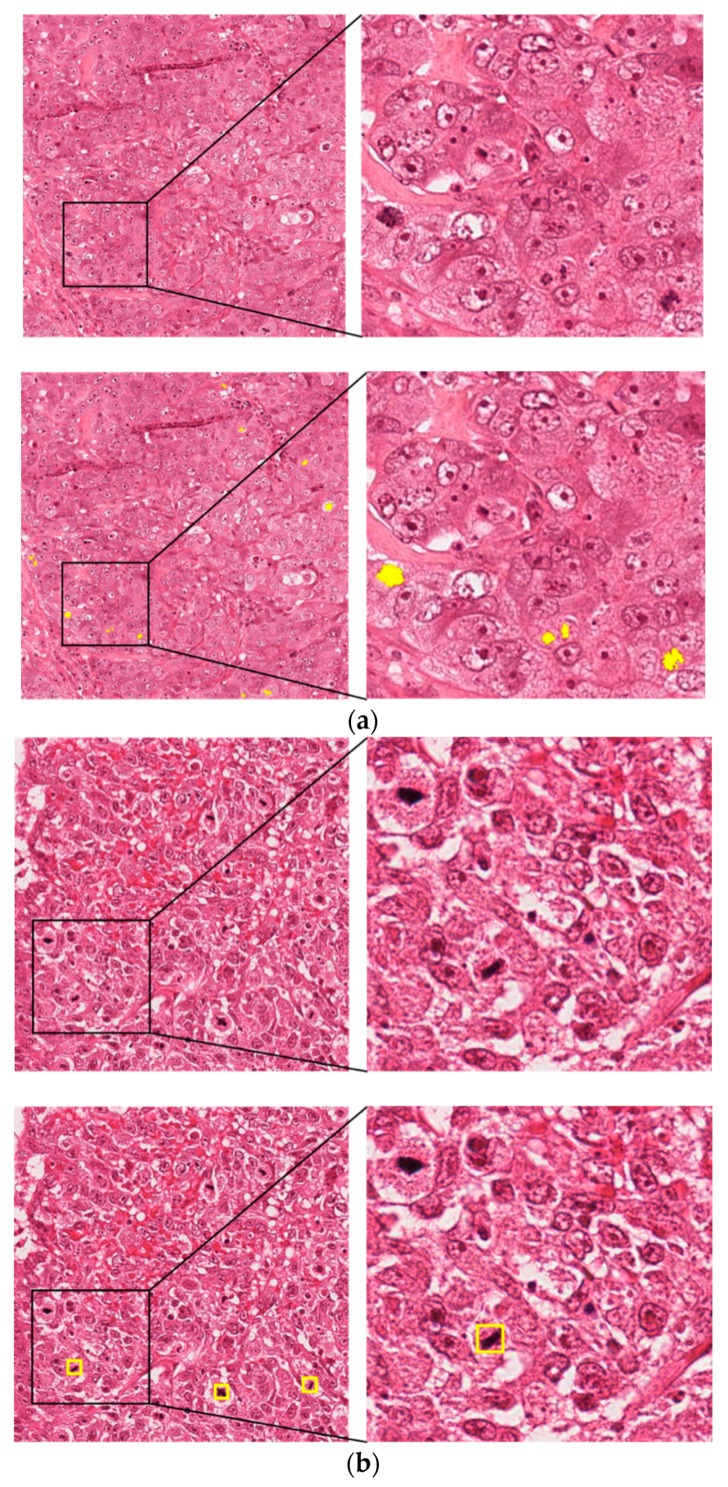
Examples of (**a**) ICPR 2012 and (**b**) ICPR 2014 datasets with ground truth images.

The ICPR 2012 MITOSIS dataset was introduced in the ICPR 2012 contest [53]. It comprises 50 RGB images of which 35 images are fixed for training and 15 images for testing. For acquiring this dataset, 10 high-power fields (HPFs) of size 512 × 512 µm^2^ at 40× magnification are selected from the biopsy images of five breast-cancer patients. The resolution of each image is 0.2456 µm per pixel, and each of the HPFs has an area of 512 × 512 µm, which implies that the image size is 2084 × 2084 pixels. Two scanners, namely, Aperio XT (scanner A) scanner and Hamamatsu NanoZoomer Scanner (scanner H) were used. Expert pathologists performed the annotations by mutual consent. There were 226 and 101 mitotic cells in the training and testing sets, respectively. In our experiments, we used Aperio XT scanner images. According to the instructions of the dataset collector, we obtained 35 images containing 226 mitotic cells in the training dataset and 15 images containing 101 mitotic cells in the testing dataset. The image dimension of 2084 × 2084 pixels was too large for training over normal GPUs. Therefore, we densely extracted patches of size 521 × 521 with a pixel difference of 40, which generates 1493 images comprising 1920 mitotic cells. Figure 3a presents sample figures of the ICPR 2012 dataset. The upper-left figure is the original dataset image, and the upper-right image presents the magnified version of a specific part in the image while the lower images are the ground truth images of the upper images.

#### 5.1.2. ICPR 2014 Dataset

The ICPR 2014 dataset was presented in the MITOS-ATYPIA-14 grand challenge [54], in which researchers were required to compete for nuclear atypia scoring and mitotic-cells count. This dataset comprised 1200 training images acquired from 16 different biopsies and 496 testing images acquired from five different breast biopsies. The size of each images is 1539 × 1376 pixels at 40× magnification, which is much smaller than those of the ICPR 2012 dataset. Pathologists annotated only the centroid pixels of each of the mitosis. This dataset comprises significant variations in the dataset images according to the tissue-acquisition process, staining, and lighting conditions, and thus, it is challenging to achieve outstanding performance. In our experiments, as the ground truths of the testing data are not provided by the organizers, we performed the experiments by splitting the training data into training and validation sets using the same split protocol mentioned in [2,24,25,27] for obtaining a fair comparison. There is no requirement of patch extraction as the image size is much smaller than that of the ICPR 2012 image. Figure 3b presents sample images from the ICPR 2014 dataset along with ground truth images.

### 5.2. Data Augmentation

Deep-learning networks usually require a sufficient amount of data for complete and efficient training; however, in the majority of cases, a large amount of data is not available. Therefore, the data augmentation technique is used to generate more data from the original data. Conventional image-processing techniques such as translation, rotation, and flipping are applied to generate new images from the original images [55]. In our experiments, we performed data augmentation only for the training of Resnet-50 and Densenet-201. For the training of Faster R-CNN, we used the original training data instead of the augmented data because sufficient training data can be obtained based on multiple patches of the input image. The detected objects by Faster R-CNN are collected from the training data and resized to 224 × 224 pixels because the input-image size for Resnet-50 and Densenet-201 pretrained on the ImageNet database should be 224 × 224 pixels. Horizontal flipping, vertical flipping, translation, and cropping-resizing operations are then applied over the image to generate an extra augmented image. We performed data augmentation by using horizontal and vertical flipping and by translation at different axes with flipping.

### 5.3. Experimental Setup and Training

#### 5.3.1. Experimental Setup

The proposed technique was implemented in MATLAB R2019a (MathWorks, Inc., Natick, MA, USA) [56] on a desktop computer with a Windows 10 operating system. The desktop computer had a central processing unit with a 3.60-GHz Intel^®^ (Santa Clara, CA, USA) Core-i7-7700 [57], 16-GB random access memory, and an NVIDIA GeForce GTX 1070 GPU [58].

#### 5.3.2. Training

In our method, training was performed at two different stages. In stage 1, we trained the Faster R-CNN, and in stage 2, Resnet-50 and Densenet-201 were trained for the score-level fusion.

##### Training of Faster R-CNN

As a feature-extraction network of Faster R-CNN, Resnet-50 was pretrained on the ImageNet database, and it was further trained with the ICPR 2012 and ICPR 2014 datasets. The end-to-end training method was used for the simultaneous training of the RPN and classification network. Several overlap ratios of bounding boxes were used to obtain better results, and the stochastic gradient descent (SGD) [59] method was used for the optimization. The SGD method efficiently optimizes all the learnable parameters of the model. The batch size, momentum, learning rate, and weight decay are 1, 0.9, 0.003, and 0.0005, respectively. The number of epochs is also a key parameter for training because the network can be under-fitted or over-fitted, and we performed the training for 25 epochs for both datasets.

##### Training of Resnet-50 and Densenet-201

For the training of Resnet-50 and Densenet-201, SGD was also used for optimization. The initial learning rate of 0.001, momentum of 0.9, learning-rate drop factor of 0.1, and mini-batch size of 50 were used for the training.

### 5.4. Performance Evaluation of Proposed Method

#### 5.4.1. Performance Evaluation Metric

The performance of the proposed techniques is measured based on the number of correctly detected mitotic cells. According to the contest criteria, a true positive is defined as a positive that exists less than 5 µm (20 pixels) and 8 µm (32 pixels), respectively, from ground truth position in the ICPR 2012 and ICPR 2014 datasets. Based on these criteria, we identify the true-positive, false-negative, and false-positive cases. A true positive indicates that the ground truth mitotic cell is correctly detected as a mitotic cell, whereas a false negative indicates that the ground truth mitotic cell is not detected as a mitotic cell. A false positive means that the ground truth non-mitotic cell is incorrectly detected as a mitotic cell. Based on these, precision, recall, and F1-measure are used for the evaluation, as shown in Equations (7)–(9).

(7) $$\mathrm{Precision}=\;\frac{TP}{TP+FP}\;,$$

(8) $$\mathrm{Recall}=\;\frac{TP}{TP+FN}\;,$$

(9) $$F1-\mathrm{measure}=\frac{2\;\mathrm{Precision}\;\mathrm{Recall}}{\mathrm{Precision}\;+\;\mathrm{Recall}}$$

where TP is the number of true positives, FP is the number of false positives, and FN is the number of false negatives.

Table 2 and Table 3 present the comparative accuracies of the proposed method with the state-of-the-art methods used with the ICPR 2012 and ICPR 2014 datasets. Our proposed technique achieved a precision, recall, and F1-measure of 0.876, 0.841, and 0.858, respectively, with the ICPR 2012 dataset, which indicates that the proposed technique outperforms all other state-of-the-art methods. The ICPR 2014 dataset is more complex and challenging as compared to the ICPR 2012 dataset. We achieved a precision, recall, and F1-measure of 0.848, 0.583, and 0.691, respectively with the ICPR 2014 dataset, which indicates that the proposed technique again outperforms all other techniques.

#### 5.4.2. Ablation Study

We present the ablation study of our proposed technique to gain the deep insights of the improvements caused by the different components in our proposed technique. Table 4 and Table 5 present the ablation study on ICPR 2012 and ICPR 2014 datasets. First, in the Faster R-CNN results, it can be observed that the highest recall is obtained by the optimization of anchor sizes and anchor scales of the Faster R-CNN. Second, post-processing impact can be seen on the improvement of precision as false positives are eliminated in the post-processing stage. Third, deep networks Resnet-50 and Densenet-201 improved the performance but were still lower than the state-of-the-art technique. Score-level fusion of these networks followed by classification has a huge impact and it can be seen that our proposed technique outperformed other state-of-the-art techniques for mitotic cell detection after combination of Faster R-CNN, post-processing and score level fusion of deep networks. Regarding the feature-driven pipeline, our method without feature-driven pipeline (FRCNN of Table 4 and Table 5) shows lower accuracy than that with feature-driven pipeline (FRCNN + PP of Table 4 and Table 5), which confirms that our feature-driven pipeline (post-processing) is necessary to enhance the accuracy with our deep learning models.

#### 5.4.3. Correct and Incorrect Detection Cases with Proposed Method

**Figure 4:**
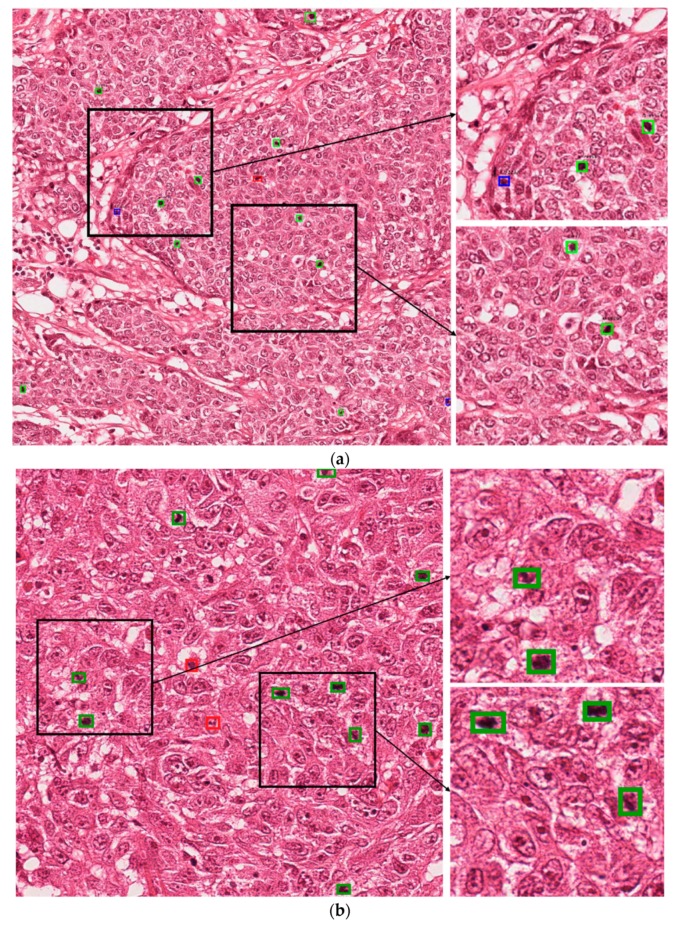
Examples of correct-detection cases of proposed method with image from (**a**) ICPR 2012 and (**b**) ICPR 2014 datasets. Green boxes indicate true positives, red boxes indicate false positives, and blue boxes indicate false negatives.

**Figure 5:**
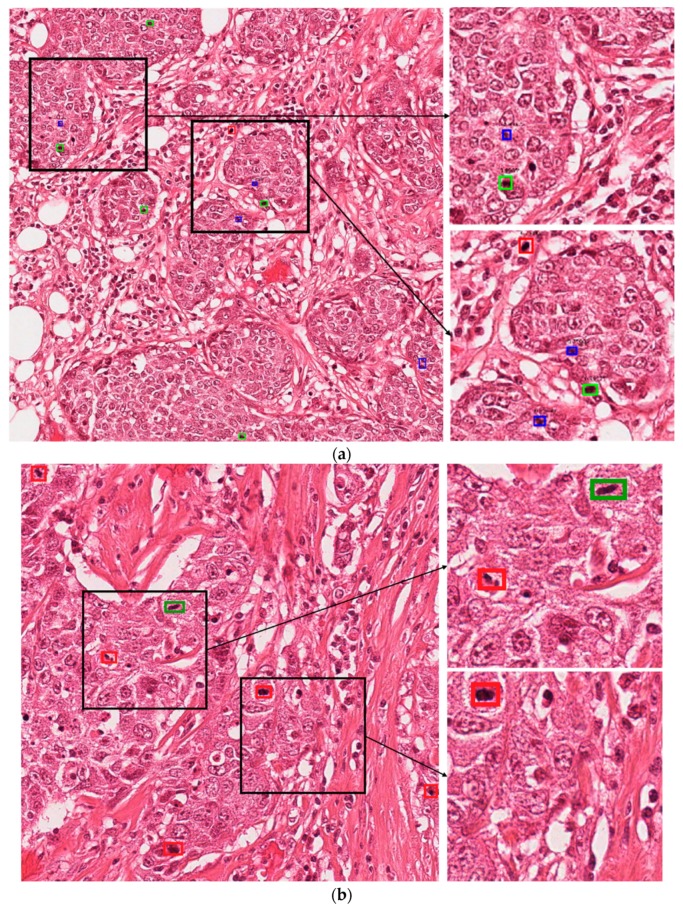
Examples of incorrect-detection cases of proposed method with image from (**a**) ICPR 2012 and (**b**) ICPR 2014 datasets. Green boxes indicate true positives, red boxes indicate false positives, and blue boxes indicate false negatives.

Figure 4 presents the correct detection cases for the proposed technique. In these figures, the green boxes represent true positives and the red box represent false positives, while the blue box represents false negatives. As shown in Figure 4, we confirm that our method can correctly detect the mitotic cells even in cases wherein it is difficult to discriminate the mitotic and non-mitotic cells based on human observation. Figure 5 presents the incorrect detection cases for the proposed technique. As shown in Figure 5, the incorrect detections mainly occur in cases where mitotic cells are either very small in size or have the same features as those of normal cells.

In clinical application, these mitotic cells are detected by a pathologist by observation of histopathology images under a high-resolution microscope. According to the Nottingham grading system, a pathologist must assign a score to three biomarkers for grading of the breast cancer. These biomarkers are tubule formation, nuclear pleomorphism and mitotic cell count [2]. A score from 1 to 3 is assigned to each of the biomarkers which are summed together for calculating the grade of breast cancer. In the case of mitotic cells, mitotic cell count from 10 high power fields are summed together and a score from 1 to 3 is assigned based on the number of mitotic cells. Score 1 is assigned for mitotic cell count 0 to 9, score 2 is assigned for mitotic cell count 10–19, and score 3 is assigned for mitotic cell count 20 or greater than 20 [61]. In the presented test images in Figure 4 and Figure 5, Figure 4a can be assigned a score of 2, while in Figure 5b, score of 1 can be assigned if a pathologist finds the grade of the breast cancer. Along with other two biomarker’s score, grade can be finalized.

#### 5.4.4. Cross-Dataset Experiment-TUPAC16

**Figure 6:**
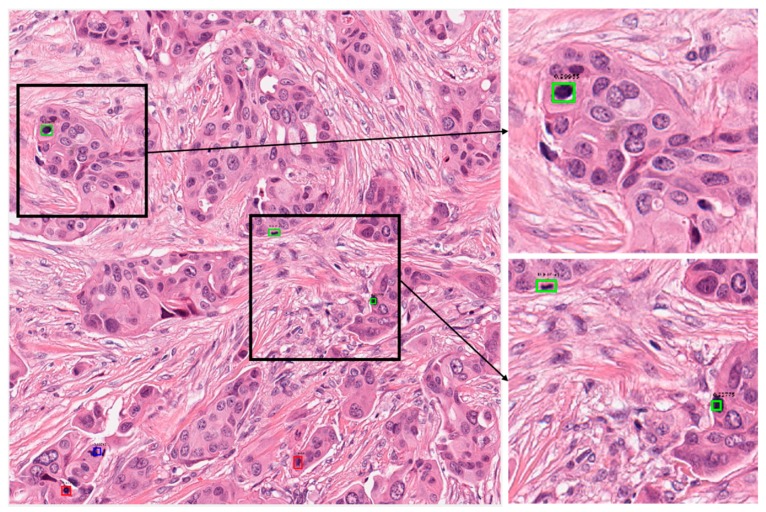
Examples of correct-detection cases of proposed method with image from TUPAC16 dataset. Green boxes indicate true positives, red boxes indicate false positives, and blue boxes indicate false negatives. TUPAC16, tumor proliferation assessment challenge 2016.

**Figure 7:**
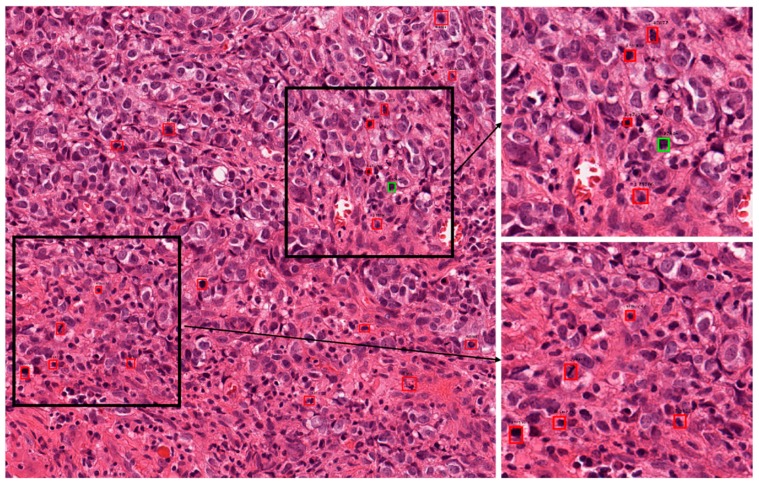
Examples of incorrect-detection cases of proposed method with image from TUPAC16 dataset. Green boxes indicate true positives, red boxes indicate false positives, and blue boxes indicate false negatives.

Automatic detection of mitotic cells is a crucial task for deep learning-based techniques. Such techniques should have a good generalization capability because cases could have variations due to tissue compositions and data acquisition environments. We tested the generalization capability of our technique on the TUPAC16 dataset. This dataset consists of cases from three pathology centers. There are 73 breast cancer cases in the train set and 34 cases in the test set. Each of the cases is represented with an area of 2 mm^2^. As the ground truths of the test data are not publicly available, we used training set images for testing the generalization capability of our proposed technique. Table 6 presents the performance of our techniques trained on ICPR2012 and tested on the TUPAC16 dataset. As shown in Table 6, our method shows a little higher accuracy than Akram et al. [62]’s method, but a little lower accuracy than Paeng et al. [63]’s method (the first ranked in TUPAC16 contest). That is because the training and testing datasets for our method are different, whereas Paeng et al. [63]’s method used the same TUPAC16 datasets for training and testing. Therefore, the results on TUPAC16 dataset proved that our proposed technique has generalization ability to the level that it can be used for real-time clinical application as shown in Table 6. There are a lot of variations in staining and tissue composition among cases, but our technique was successful in detection as shown in Figure 6 and Figure 7 where correct and incorrect detections are presented by the proposed technique. It can be observed that our technique had less performance than the state-of-the-art results, but we can state that our technique has a good generalization capability and can be improved further by tuning and training with the TUPAC16 dataset.

## 6. Activation Maps and Discussion

**Figure 8:**
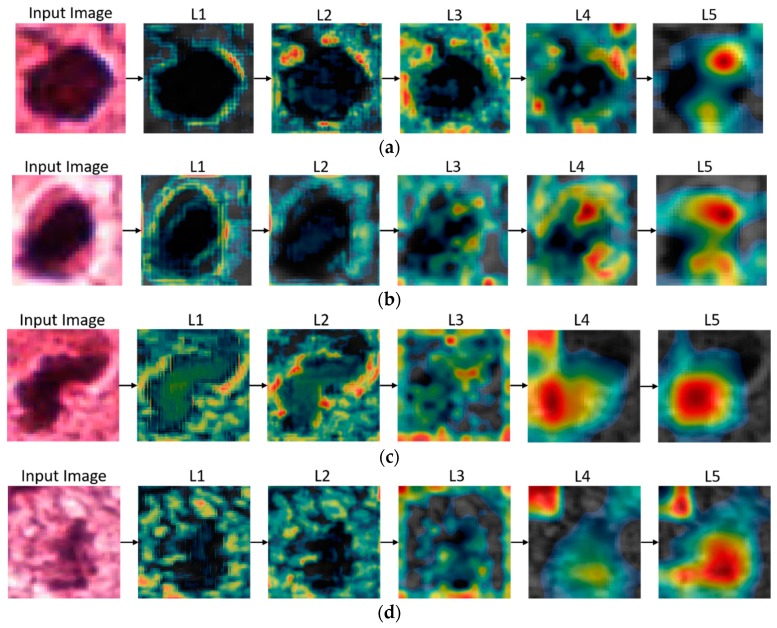
Obtained activation maps from different parts of (**a**), (**b**) Resnet-50, and (**c**), (**d**) Densenet-201 with mitotic and non-mitotic cell images. (a) and (c) comprise mitotic cells whereas (b) and (d) comprise non-mitotic cells. In (a) and (b), L1–L5 are the Resnet-50 layers Conv2-1, Conv3-4, Conv4-1, Conv4-6, and Conv5-3, respectively, as presented in Table A1, whereas L1–L5 in (c) and (d) are the Densnet-201 layers Convolution (1), Dense Block (1), Dense Block (2), Dense Block (3), and Dense Block (4), respectively.

Deep-learning networks are often considered as “black boxes” because there is no clear explanation behind a specific decision. Hundreds of features are learned during training, which are then used for the decision making. The use of class-specific discriminative regions (activation maps) is one technique that can be used for the investigation of a network to identify the regions of images that are responsible for the decision making. Figure 8 presents the class-specific discriminative regions, also known as activation maps of an image when it passes through the different layers of our trained Resnet-50 and Densenet-201 classifiers. For presentation purpose, we consider the average activation map of a layer, followed by its representation in a pseudo-color scheme, in which red indicates the maximum value and blue indicates the minimum value. In detail, since all the layers of the networks are multi-dimensional along the feature channel axis, for presentation purpose we took the average of the activation maps along the feature channel axes of a layer. This way, we present a single image for a layer. Using these activation maps, we can obtain a visual explanation of the regions of an image that contribute the most in the decision making. It can be easily observed from Figure 8 that although there is no visible difference between the presented cases of mitotic and non-mitotic cells, our trained networks still find deep features to differentiate between the two cells. The earlier layers learn simple features such as color and edges, and the deeper layers learn complex features that have a significant impact on decision making. Moreover, we identified from class-activation maps that our training is not biased towards wrong ROIs. Moreover, as shown in Figure 8, there is noticeable characteristics in activation maps in the case of mitotic cells as compared to those of non-mitotic cells. That is, multiple regions are activated in case of non-mitotic cells, which implies that our trained models have the ability to successfully differentiate between mitotic and non-mitotic cells.

The key observations made in this work are as follows:

- Our proposed-technique results show that recent advances in deep-learning algorithms have decreased the gap between diagnoses performed by human experts and computers. Moreover, a good performance with the ICPR 2012 and ICPR 2014 datasets has proved the generalization capabilities of our proposed technique, and thus, our technique may be used for various lesion detections.
- We have observed that significant variation exists in the sizes of the mitotic cells. Therefore, Faster R-CNN feature-extraction network and anchor-boxes selection play a key role in the detection of mitotic cells. By using Resnet-50 for feature extraction, we successfully extracted efficient features because Resnet-50 uses skip connections, and thus, the mitotic cell’s information is not lost. Moreover, we fixed the anchor scale size to 64 instead of 128, 256, or 512 and selected anchor boxes that have an intersection-over-union value less than or equal to 0.5 with ground truths. Therefore, by using Resnet-50 as a feature-extraction network, fixing the anchor-scale size to 64, and limiting the number of anchor boxes, we achieved the state-of-the-art performance.
- We have also observed that Faster R-CNN also depends on the underlying feature-extraction network and RPN. Therefore, in our case Faster R-CNN rapidly converges in only 25 epochs, because of the use of Resnet-50 as a feature-extraction network, the smaller anchor scale, and the limited anchor boxes.
- We have observed that some of the false-positive cases comprise an irregular morphology and dark bluish color and have large variations in texture. These issues can be eliminated by using handcrafted features such as LBP, HOG, and statistical and color features for improving the performance.
- Mitotic-cell-detection techniques [2,24,25,26,27] comprise the use of additional classifiers for performance improvement. Although classifiers such as Resnet-50 and Densenet-201 exhibit an outstanding performance owing to Resnet-50′s residual learning and skip connection for feature reusability and Densenet-201′s feature propagation, feature reusability, and smaller number of parameters, we still observed that the performance can be further improved because single-modality data lack uniqueness and universality. Therefore, in our proposed technique we performed score-level fusion and improved our obtained results as compared to those of state-of-the-art methods.
- Deep-learning networks require a large amount of data for successful training. Owing to the lack of data, some other techniques such as data augmentation are used to increase the data. Data augmentation in the case of mitotic-cell detection is a challenging problem because there are minute differences between mitotic and non-mitotic cells. We observed that the flipping and translation technique for data augmentation produces robust data as proved by the activation maps in Figure 8, where trained classifiers successfully found features in the test data for decision making.

In general, pathologists examine the color and morphological appearances under high-resolution microscopes in order to differentiate between the mitotic and non-mitotic cells. Although pathologists are superior in decision making but these procedures are tiresome and tedious due to extensive similarities among cells. Therefore, AI-based techniques can assist pathologists by pointing out the positive cases. Different AI-based techniques are used to assist pathologists. Our proposed technique can also be used to assist pathologists because we have outperformed the existing techniques for mitotic-cells detection by using a combination of Faster R-CNN, post-processing, and score-level fusion.

## 7. Conclusions

In this paper, we presented a multi-stage mitotic-cell-detection technique based on Faster-RCNN, post-processing, and deep CNNs. Faster RCNN performs the initial detection in stage 1, which is further improved by post-processing and score-level fusion of the deep CNNs in stages 2 and 3, respectively. The performance thus obtained comprises the combined detections of all the stages. The strengths of this work comprise the adoption of Resnet-50 for the feature extraction in the Faster R-CNN, extraction of appropriate features in the post-processing, and score-level fusion of the Resnet-50 and Densenet-201 classifiers. Moreover, our proposed technique outperforms all the existing state-of-the-art techniques on the two open databases of ICPR 2012 and ICPR 2014. This proposed technique can be used for developing an automatic grading system that could serve as a second-opinion system for pathologists. It can also highlight cases that require special attention. This research is useful for pathologists and researchers working in the field of cancer diagnosis based on histology images. Future research work can be based on our publicly available trained models, and a fair comparison of results is also possible.

In the future, we intend to improve the accuracies and decrease the computational cost of the presented technique by developing a customized network. We intend to validate our technique using larger databases that provide a diversity of breast cancer cases. We also plan to increase the scope of this technique to applications that combine the biomarkers of nuclear atypia, tubule formation, and mitotic-cell count for the diagnosis and grading of breast cancers.

## Figures and Tables

**Table 1:** Comparison of previous studies and proposed method on mitosis detection.

Category	Method	Datasets	Strength	Weakness
Hand-crafted features	Morphological and statistical features with decision tree classifier [12]	ICPR 2012	Efficient in capturing texture features for mitotic cell segmentation	Low detection performance and computationally expensive
	LBP and SVM classifier [13]	ICPR 2012	High discriminative power, computational simplicity, and invariance to grayscale changes	Affected by rotation and limited structural information capturing
	Shape, texture, and intensity features with SVM classifier [14]	ICPR 2012	Small amount of parameter tuning and low user effort	Low detection performance and object segmentation using open-source software
	Intensity, texture, and regenerative random forest tree classifier [15]	ICPR 2012	Good performance for large data	Computationally expensive and complex due to random forest tree
Deep features	Sliding-window-based classification [17]	ICPR 2012	Good detection performance	Computationally expensive
	Combination of color, texture, and shape features, and CNN features with SVM classifier [18]	ICPR 2012	Easy to accommodate for multi-scanner data without major redesign	Computationally expensive
	Handcrafted and CNN features, random forest classifier, and CNN [19]	ICPR 2012	Fast and high precision	Using fixed global and local threshold in object-detection stage
	FCN model for objects segmentation and CNN for classification [20]	ICPR 2012	Robust, fast, and high precision	Not suitable for weakly annotated datasets, and object detection stage is computationally expensive
	Faster R-CNN-based detection and Resnet-50 for classification [2]	ICPR 2012 ICPR 2014	Good performance and inference time	VGG-16 is used as a feature extraction network of Faster R-CNN, which have the vanishing gradient issue
	Concentric circle approach for objects detection and FCN for segmentation [24]	ICPR 2012 ICPR 2014 TUPAC-16	Good technique for weakly annotated datasets	Low detection performance
	Modified Faster R-CNN with Resnet-101 feature-extraction network [25]	ICPR 2014 TUPAC-16	Less inference time	Resnet-101 can be replaced by shallow network
	Lightweight region-based R-CNN [26]	ICPR 2012 ICPR 2014	No requirement of powerful GPUs	Low detection performance
	Mask R-CNN for object detection and handcrafted and CNN features [27]	ICPR 2012 ICPR 2014	Highest performance and inference time	Using expensive GPUs and intensive training
	Faster R-CNN and score-level fusion of Resnet-50 and Densenet-201 (proposed)	ICPR 2012 ICPR 2014	High detection performance	Long processing time owing to multiple networks and intensive training

ICPR, international conference on pattern recognition; LBP, local binary pattern; SVM, support vector machine; CNN, convolutional neural network; Faster R-CNN, Faster region convolutional neural network; TUPAC, tumor proliferation assessment challenge; VGG, visual geometry group.

**Table 2:** Comparisons of the proposed method and previous techniques with ICPR 2012 dataset.

Technique	Precision	Recall	F1-Measure
Sommer et al. [14]	0.519	0.798	0.629
Malon et al. [18]	0.747	0.590	0.659
Tashk et al. [13,60]	0.699	0.72	0.709
Irshad [12,60]	0.698	0.74	0.718
Wang et al. [19]	0.84	0.65	0.735
Ciresan et al. [17]	0.88	0.70	0.782
Li et al. [26]	0.78	0.79	0.784
Chen et al. [20]	0.804	0.772	0.788
Li et al. [24]	0.846	0.762	0.802
Paul et al. [15]	0.835	0.811	0.823
Li et al. [2]	0.854	0.812	0.832
Proposed method	0.876	0.841	0.858

**Table 3:** Comparisons of the proposed method and previous techniques with ICPR 2014 dataset (*N.R.* means “not reported”).

Technique	Precision	Recall	F1-Measure
Li et al. [2]	*N.R.*	*N.R.*	0.572
Cai et al. [25]	0.53	0.66	0.585
Li et al. [24]	0.495	0.785	0.607
Li et al. [26]	0.654	0.663	0.659
Dodballapur et al. [27]	0.58	0.82	0.68
Proposed method	0.848	0.583	0.691

**Table 4:** Quantitative comparison of each component of the proposed method on ICPR 2012 dataset. (FRCNN indicates Faster R-CNN, PP indicates post-processing (feature-driven method), D-net indicates Densenet-201, R-net indicates Resnet-50 and SF indicates score-level fusion of Densent-201 and Resnet-50.)

Technique	Precision	Recall	F1-Measure
FRCNN	0.540	0.851	0.661
FRCNN + PP	0.641	0.851	0.731
FRCNN + PP + D-net	0.793	0.722	0.756
FRCNN + PP + R-net	0.7692	0.792	0.780
FRCNN + PP + SF (Proposed)	0.876	0.841	0.858

**Table 5:** Quantitative comparison of each component of the proposed method on ICPR 2014 dataset. (FRCNN indicates Faster R-CNN, PP indicates post-processing (feature-driven method), D-net indicates Densenet-201, R-net indicates Resnet-50 and SF indicates score-level fusion of Densent-201 and Resnet-50.)

Technique	Precision	Recall	F1-Measure
FRCNN	0.521	0.641	0.575
FRCNN + PP	0.536	0.64	0.584
FRCNN + PP + D-net	0.674	0.599	0.634
FRCNN + PP + R-net	0.689	0.586	0.633
FRCNN + PP + SF (Proposed)	0.848	0.583	0.691

**Table 6:** Comparisons of the proposed method on cross-dataset TUPAC16 with ICPR 2012 dataset trained networks (TUPAC16, tumor proliferation assessment challenge 2016. N.R. means “not reported” and proposed method-12 indicates networks trained on ICPR2012 dataset).

Technique	Precision	Recall	F1-Measure
Akram et al. [62]	0.61	0.67	0.64
Paeng et al. [63]	*N.R.*	*N.R.*	0.652
Proposed method-12	0.641	0.642	0.642

## Appendix A

**Table A1:** Resnet-50 feature-extraction network (Conv means convolutional layer)**.**

Layer Type		Output Size	Numbers of Filters	Kernel Size	Strides	Paddings	Iterations
Image input layer		224 × 224 × 3					
Conv1		112 × 112 × 64	64	7 × 7 × 3	2	3	1
Maximum pool		55 × 55 × 64	1	3 × 3	2	0	1
Conv2	Conv2-1 (1 × 1 Convolutional Mapping)	55 × 55 × 64	64	1 × 1 × 64	1	0	1
		55 × 55 × 64	64	3 × 3 × 64	1	1	
		55 × 55 × 256	256	1 × 1 × 64	1	0	
		55 × 55 × 256	256	1 × 1 × 64	1	0	
	Conv2-2-Conv2-3 (Identity Mapping)	55 × 55 × 64	64	1 × 1 × 256	1	0	2
		55 × 55 × 64	64	3 × 3 × 64	1	1	
		55 × 55 × 256	256	1 × 1 × 64	1	0	
Conv3	Conv3-1 (1 × 1 Convolutional Mapping)	28 × 28 × 128	128	1 × 1 × 256	2	0	1
		28 × 28 × 128	128	3 × 3 × 128	1	1	
		28 × 28 × 512	512	1 × 1 × 128	1	0	
		28 × 28 × 512	512	1 × 1 × 256	2	0	
	Conv3-2-Conv3-4 (Identity Mapping	28 × 28 × 128	128	1 × 1 × 512	1	0	3
		28 × 28 × 128	128	3 × 3 × 128	1	1	
		28×28×512	512	1 × 1 × 128	1	0	
Conv4	Conv4-1 (1 × 1 Convolutional Mapping)	14 × 14 × 256	256	1 × 1 × 512	2	0	1
		14 × 14 × 256	256	1 × 1 × 512	1	1	
		14 × 14 × 1024	1024	1 × 1 × 512	1	0	
		14 × 14 × 1024	1024	1 × 1 × 512	2	0	
	Conv4-2-Conv4-6 (Identity Mapping)	14 × 14 × 256	256	1 × 1 × 1024	1	0	5
		14 × 14 × 256	256	1 × 1 × 256	1	1	
		14 × 14 × 1024	1024	1 × 1 × 256	1	0	
Conv5	Conv5-1 (1 × 1 Convolutional Mapping)	7 × 7 × 512	512	1 × 1 × 1024	2	0	1
		7 × 7 × 512	512	3 × 3 × 512	1	1	
		7 × 7 × 2048	2048	1 × 1 × 512	1	0	
		7 × 7 × 2048	2048	1 × 1 × 1024	2	0	
	Conv5-2-Conv5-3 (Identity Mapping)	7 × 7 × 512	512	1 × 1 × 2048	1	0	2
		7 × 7 × 512	512	3 × 3 × 512	1	1	
		7 × 7 × 2048	2048	1 × 1 × 512	1	0	

**Table A2:** Region proposal network architecture (CL indicates convolutional layer)**.**

Layer Type	Number of Filters	Output Size	Kernel Size	Strides	Paddings
5_3rd CL Input layer		7 × 7 × 2048			
6th CL (ReLU)	512	7 × 7 × 2048	3 × 3 × 512	1	1
Classification CL (Softmax)	18	7 × 7 × 18	1 × 1 × 512	1	0
6th CL Regression CL	36	7 × 7 × 36	1 × 1 × 512	1	0

**Table A3:** Classification network (ROI coordinate* comprises x_min, y_min, x_max, and y_max of ROI of each proposal.

Layer Type	Output Size
5_3rd CL RPN proposal region Input layer	7 × 7 × 2048 (height × width × depth) 300 × 4 (ROI coordinate *)
ROI pooling layer	7 × 7 × 512 (height × width × depth) × 300
1st fully connected layer (ReLU) (Dropout)	4096 × 300
2nd fully connected layer (ReLU) (Dropout)	4096 × 300
Classification convolutional layer (Softmax)	2 × 300
2nd Fully connected layer Regression fully connected layer	4 × 300

RPN, region proposal network.
